# A Literature Review of Major Clinical Trials That Contributed to Treatment Protocols of Irritable Bowel Syndrome With Diarrhea

**DOI:** 10.7759/cureus.26092

**Published:** 2022-06-19

**Authors:** Hassan Elsaygh, Iman Alhafez, Mohammed Jaber, Maice Al-Rasheed, Anas Zaher, Jude ElSaygh

**Affiliations:** 1 Internal Medicine, University of Debrecen, Debrecen, HUN

**Keywords:** chronic abdominal pain, ibs, treatment, irritable bowel syndrome, inflammation, diarrhea, irritable bowel disease

## Abstract

The most common reason for seeing a gastroenterologist is irritable bowel syndrome (IBS). IBS was thought to be a functional disease; however, there are now numerous alternative pathophysiologic pathways that can explain the symptoms. The pathophysiology of IBS is diverse and not well understood. Most current first-line treatments for IBS target the primary symptom and mainly impact one symptom in the symptom complex. The purpose of this study was to summarize the data on new medicines used to treat IBS. We conducted a bibliographic search in Google Scholar and PubMed focused on medication clinical trials in IBS with diarrhea. New medications for IBS with diarrhea target important pathways in the pathophysiology of these disorders, improving both the abnormal bowel habit and other significant symptoms such as abdominal pain and bloating.

## Introduction and background

Irritable bowel syndrome (IBS) is a prevalent, costly, and potentially severe functional gastrointestinal illness, marked by recurring abdominal pain and changes in bowel habits [1]. The global prevalence of IBS has been estimated to be 11% [2]. IBS has been linked to psychological issues, including depression, anxiety, and somatization. For the clinical diagnosis of IBS, the Rome IV criteria are widely utilized. To meet the criteria, one must have had recurring abdominal discomfort for at least one day per week in the previous three months and two or more of the following symptoms: defecation, a change in stool frequency, and a change in stool appearance [1,3].

There are three forms of IBS: constipation-predominant IBS (IBS-C), diarrhea-predominant IBS (IBS-D), and mixed-IBS (IBS-M). While patients with IBS-C or IBS-D have constipation or diarrhea, people with IBS-M may experience both constipation and diarrhea on most days. Regardless of the kind of IBS, each is frequently linked with additional symptoms such as flatulence, a feeling of incomplete evacuation, or abdominal pain. Patients with IBS may have a lower quality of life and productivity as a result of the disease's burden [4]. Even though IBS is a prevalent disease, frequently chronic and recurring in nature, the underlying pathophysiology of IBS is still unknown [1]. There are few effective treatment options available, and most of them focus on particular symptoms rather than the entire disease burden. This approach is partly due to a lack of knowledge about the underlying pathophysiology of these conditions, as well as the relative importance of existing pathophysiological elements for particular patients. IBS is currently thought to be a disorder of disrupted gut-brain interactions with anomalies at several points along the gut-brain axis [5], such as altered gastrointestinal motility, visceral hypersensitivity, increased intestinal permeability, immunological activation, and altered gut flora. All of these anomalies could be used as therapeutic targets in the future [5].

IBS appears to be more common in females in most societies, and it tends to diminish with age. It clusters in families, but studies assessing the association of IBS with socioeconomic status show conflicting results [5].

Treatment options for IBS include dietary and lifestyle changes, as well as psychological and pharmaceutical interventions. Because of the diverse presentation of IBS and the inadequate data supporting the efficacy of many of the treatment choices, there is no clear first-line treatment for all people with IBS. When it comes to controlling IBS, the decision between one treatment modality and another is frequently the first of many. If drugs are chosen as the therapy modality, the next problem is determining which medication to use, followed by ensuring that the medication is used optimally in patients [4].

This review focuses on new and emerging treatment options for IBS, including efficacy and adverse effects in patients.

## Review

Irritable bowel syndrome with diarrhea (IBS-D) rifaximin re-treatment study (TARGET3)

This study evaluated the effectiveness and safety of repeat treatment with rifaximin 550 mg three times a day (TID) in patients with IBS-D who responded to initial treatment with rifaximin 550 mg TID. The study enrolled 2583 participants and included a 10 (±3) day run-in treatment period, during which subjects received a single-blind placebo and completed a daily IBS symptom diary. Subjects who had active symptoms of IBS-D and satisfied other entry criteria entered the open-label period of study. The duration of the trial was two years and three months. It was a randomized, parallel assignment, double-masked study. The inclusion criteria included IBS confirmed by the Rome III diagnostic criteria, at least 18 years of age, colonoscopy within the past 10 years to rule out inflammatory bowel disease, and a willingness to maintain a stable diet. In the first arm of the study, 2583 subjects received open-label rifaximin 550 mg TID for two weeks, with a four-week treatment-free follow-up. Responders continued into maintenance phase 1 (treatment-free). Non-responders withdrew from the study. In the second arm, 328 subjects received rifaximin in the open-label period, eventually met the criteria for recurrence, and were randomized to the rifaximin group for the double-blind retreatment period. In the third arm, 308 subjects received rifaximin in the open-label period, eventually met the criteria for recurrence, and were randomized to the placebo group for the double-blind retreatment period. The primary outcome measured was the number of repeat treatment responders.

The results showed that the proportion of patients who responded to repeat treatment during the four-week treatment-free follow-up in the first double-blind phase was 32.6% for the rifaximin group and 25.0% for the placebo group (p = 0.0232).

In terms of adverse events (AEs), no deaths were noted in any of the groups. In the open-label rifaximin arm, 1.09% of participants experienced AEs, the most prevalent being gastrointestinal disorders, non-cardiac chest pain, intervertebral disc degeneration, and infections. In the double-blind rifaximin arm, 1.22% of participants experienced AEs, which included *Clostridium difficile* colitis, breast cancer, and dyspnoea. Regarding the double-blind placebo arm, 1.30% of participants experienced AEs, which included coronary artery occlusion, non-cardiac chest pain, transient ischemic attack, and hypertension [6].

Rifaximin 3 times/day (TID) for non-constipation irritable bowel syndrome (IBS) (TARGET 1)

This study evaluates the efficacy of a 14-day course of rifaximin given three times a day vs. placebo in providing adequate relief of IBS symptoms. The study enrolled 623 participants. The duration of the trial was one year. It was a randomized, parallel assignment, quadruple-masked study. The inclusion criteria included confirmed IBS diagnosis per the Rome II criteria for the diagnosis of IBS, colonoscopy within two years as part of the IBS diagnostic evaluation, and active symptoms of non-constipation IBS at baseline. The two arms of the study consisted of 292 participants given a placebo tablet and 283 recipients of rifaximin 550 mg tablets. Both groups took the tablets three times daily for two weeks and were followed for 10 weeks after completion of the treatment period. The primary outcome was measured as the proportion of subjects who had adequate relief of global IBS symptoms for at least two of the four weeks during the primary evaluation period.

The results showed that the proportion of subjects who had relief from global IBS symptoms for at least two of the four weeks was 31.2% for the placebo group and 40.8% for the rifaximin group (p = 0.01). Among the secondary outcomes measured, those who had relief of IBS-related bloating for at least two of the four weeks were 28.7% for the placebo group and 39.5% for the rifaximin group (p = 0.005).

In terms of AEs, there were no deaths noted in either group. In the placebo group, eight out of 314 participants experienced AEs, the most predominant being gastrointestinal and hepatobiliary disorders, labile hypertension, amnesia, and schizophrenia of the paranoid type. In the rifaximin group, three out of 309 participants experienced AEs, including costochondritis, alcohol withdrawal syndrome, and a road traffic accident. Both groups reported gastrointestinal disorders, infections, infestations, and musculoskeletal, connective tissue, nervous system, respiratory, thoracic, and mediastinal disorders [7].

Rifaximin 3 times/day (TID) for non-constipation irritable bowel syndrome (IBS) (TARGET 2)

This study evaluated the efficacy of a 14-day course of rifaximin given three times a day vs. placebo in providing adequate relief of IBS symptoms. The study enrolled 637 participants. The duration of the trial was one year. It was a randomized, parallel assignment, quadruple-masked trial. The inclusion criteria included confirmed IBS diagnosis per the Rome II criteria for the diagnosis of IBS, colonoscopy within two years as part of the IBS diagnostic evaluation, and active symptoms of non-constipation IBS at baseline. The two arms of the study had 302 participants given placebo tablets and 301 participants given rifaximin 550 mg tablets. Both groups took the tablets three times daily for two weeks and were followed for 10 weeks after completion of the treatment period. The primary outcome measured was the proportion of subjects who had adequate relief of global IBS symptoms for at least two of the four weeks during the primary evaluation period.

The results showed the proportion of subjects who had adequate relief of global IBS symptoms for at least two of the four weeks was 32.2% for the placebo group and 40.6% for the rifaximin group (p = 0.03). Among the secondary outcomes measured, those who had relief of IBS-related bloating for at least two of the four weeks were 31.9% in the placebo group and 41.0% in the rifaximin group (p = 0.02).

In terms of AEs, no deaths were noted in both groups. In the placebo group, seven out of the 320 participants experienced AEs, the most prominent being chest pain, appendicitis, metabolic acidosis, musculoskeletal and connective tissue disorders, psychiatric disorders, and asthma. In the rifaximin group, seven out of 315 participants reported AEs, including atrial fibrillation, chest pain, non-cardiac chest pain, meningitis, intervertebral disc displacement, breast cancer, hypoesthesia, and hypertension. Both groups reported gastrointestinal disorders, musculoskeletal disorders, connective tissue disorders, nervous system disorders, infections, and infestations [8].

12-week efficacy and safety study of ibodutant in women with irritable bowel syndrome with diarrhea (IBS-D) (IRIS-3)

This trial aims to evaluate the efficacy and safety of oral ibodutant 10 mg once daily as compared to a placebo in women with IBS-D over a 12-week treatment period. A weekly response for abdominal pain intensity and stool consistency is recorded for at least 50% of the treatment weeks. The study enrolled 535 participants, in which the first patient was screened on February 27, 2014, and the first patient was randomized on March 21, 2014. The last patient completed the study on June 22, 2015. A total of 1237 patients entered a two-week screening period, 1034 entered the qualifying two-week run-in period, and 535 of them were randomized. After completion of the double-blind 12-week treatment, 453 patients entered the randomized withdrawal (RW) period. The duration of the trial was one year. It was a randomized, parallel assignment, quadruple-masked study. The inclusion criteria included female patients aged 18 years or older with a clinical diagnosis of IBS-D according to the symptoms-based criteria as per the Rome III modular questionnaire criteria. The first arm of the study included 227 participants given ibodutant 10 mg tablets who were re-randomized at week 13 in a 1:1 ratio to either ibodutant 10 mg or placebo for an additional four weeks of treatment. The second arm of the study included 225 participants who were given placebo tablets and were mock-re-randomized (switched in blinded conditions) to ibodutant at week 13 for an additional four weeks of treatment. In both arms of the study, the oral tablets were given once daily for 12 weeks of treatment. The primary outcome measured the weekly response of abdominal pain intensity and stool consistency over 12 weeks of treatment in at least 50% of the weeks of treatment.

The results showed that the weekly response for abdominal pain intensity and stool consistency over 12 weeks of treatment in at least 50% of the weeks was 35.7% in the ibodutant 10 mg arm and 34.7% in the placebo arm. The secondary outcome measured the weekly response for abdominal pain alone over 12 weeks of treatment in at least 50% of the weeks, which was 48.0% in the ibodutant 10 mg arm versus 47.7% in the placebo arm.

In terms of AEs, no deaths were noted in both groups. Two out of 264 participants in the placebo arm experienced AEs, including atrial fibrillation and circulatory collapse. Both groups reported gastrointestinal disorders, infections, infestations, musculoskeletal, connective tissue, nervous system, respiratory, thoracic, and mediastinal disorders [9].

Ibodutant for relief of irritable bowel syndrome with diarrhea (IBS-D) (IRIS-2)

The study evaluated the efficacy and safety of three doses of ibodutant, given once daily for eight weeks versus placebo in IBS-D patients. Efficacy is evaluated in terms of overall symptom relief and abdominal pain/discomfort relief after eight weeks of treatment. The study enrolled 565 participants at 78 study sites in eight European countries. A run-in period was planned to confirm eligibility criteria. Because of the high screen fail rate, it was impossible to predict how many patients would be enrolled. Although the enrollment goal was reached, five patients in screening continued the study. The duration of the trial was one year and six months. It was a randomized, parallel assignment, triple-masked study. The inclusion criteria included male or female patients aged 18-70 years with a clinical diagnosis of IBS-D according to the Rome III criteria and recurrent abdominal pain for at least three days per month in the last three months. The four arms of the study included 130 participants given ibodutant 1 mg, 131 participants given ibodutant 3 mg, 133 participants given ibodutant 10 mg, and 133 participants given placebo oral tablets once daily. The primary outcome measured the response to the relief of overall IBS symptoms and of abdominal pain/discomfort at the end of eight weeks of treatment, where the response is defined as at least six weeks of satisfactory relief during eight weeks of treatment (75% rule).

The results showed that the number of responders for relief at the end of eight weeks of treatment according to the 75% rule was 45 participants in the ibodutant 1 mg arm, 46 participants in the ibodutant 3 mg arm, 55 participants in the ibodutant 10 mg arm, and 39 participants in the placebo arm (p < 0.05). Among the secondary outcomes measured, the number of responders to relief, defined as at least four weeks during eight weeks of treatment (50% rule), was 72 participants in the ibodutant 1 mg arm, 61 in the ibodutant 3 mg arm, 74 in the ibodutant 10 mg arm, and 55 in the placebo arm (p < 0.05).

In terms of AEs, no deaths were noted in any of the groups. Among the AEs, one out of 142 participants in the Ibodutant 3 mg arm experienced acute appendicitis, one out of 139 participants in the Ibodutant 10 mg arm experienced type 2 diabetes mellitus, and three out of 143 participants in the placebo arm experienced events including mydriasis, worsened abdominal pain, and uterus myomatosus. All the groups reported gastrointestinal disorders, infections, infestations, blood creatine, and nervous system disorders, with the exception that there was no blood creatine reported in the ibodutant 3 mg arm [10].

Efficacy of eluxadoline in the treatment of irritable bowel syndrome with diarrhea in patients with inadequate control of symptoms with prior loperamide use

This study evaluated the efficacy and safety of eluxadoline 100 mg twice a day (BID) versus placebo for the treatment of patients with IBS-D who reported the use of loperamide in the prior 12 months failed to provide control of their IBS-D symptoms. The study enrolled 346 participants, of which 344 participants received at least one dose of the study drug, which comprises the safety population. Randomization and treatment assignment were based on a randomization scheme prepared by Allergan Biostatistics prior to the start of the study. The duration of the trial was one year. It was a randomized, parallel assignment, quadruple-masked study. The inclusion criteria included patients with a diagnosis of IBS-D, defined by the Rome III criteria as loose (mushy) or watery stools ≥25% and hard or lumpy stools ≤25% of bowel movements, and those who had a colonoscopy performed within five years prior to screening if they are at least 50 years of age. The two arms of the study were 146 participants given eluxadoline 100 mg oral tablets twice daily (BID) with food and 149 participants given placebo-matched eluxadoline oral tablets BID with food for 12 weeks. The primary outcome measured the percentage of participants who were composite responders based on improvements from baseline in daily worst abdominal pain and daily stool consistency scores.

The results showed that the percentage of participants who were composite responders from baseline to 12 weeks was 22.7% for the eluxadoline 100 mg group and 10.3% for the placebo group (p = 0.0022). Among the secondary outcomes measured, the percentage of stool consistency responders at week 12 was 27.9% for the eluxadoline 100 mg group and 16.7% for the placebo group (p = 0.0119).

In terms of AEs, no deaths were noted in both groups. Among the AEs, one out of 171 participants in the eluxadoline 100 mg arm experienced a pancreatic mass. Three out of 173 participants in the placebo group experienced AEs, including cardiac failure congestive, cellulitis, pneumonia, uterine leiomyoma, reproductive system, and breast disorders. Both groups reported nausea [11].

Eluxadoline bile acid malabsorption (BAM) study

This study evaluated the possibility of a differential effect of eluxadoline on altered bowel function in IBS-D participants with and without evidence of bile acid malabsorption (BAM). The study included 24 participants who were enrolled in a four-week treatment period where 12 of them had evidence of BAM and 12 had no evidence of BAM. The duration of the study was two years. It was a non-randomized, parallel assignment study. The inclusion criteria included adults aged 18-75 years with a diagnosis of IBS-D per the Rome IV criteria, fasting serum 7a-hydroxy-4-cholesten-3-one (7αC4) level ≥ 52.5 ng/mL or total fecal bile acid (BA) > 2337 micromoles/48 hours, completed the electronic diary (eDiary) on ≥ 10 of the 14 days prior to day one, and not used loperamide rescue medication on > three of the 14 days prior to day one. The first arm of the study included IBS-D participants with evidence of BAM who were treated with eluxadoline 100 mg oral tablets BID with food for four weeks. The second arm included IBS-D patients without evidence of BAM who were treated with eluxadoline 100 mg oral tablets BID with food for four weeks. The primary outcome measure was the change from baseline in the average Bristol Stool Form Scale (BSFS) score over the four-week treatment period.

The results in both arms of the study showed a negative change from baseline in average (BSFS), which indicates improvement as a result of treatment. When assessing the number of participants who experienced potentially clinically significant changes in laboratory tests, the first arm of the study in which participants had evidence of BAM showed no change in laboratory tests. In the second arm of the study, in which participants had no evidence of BAM, two participants showed changes in laboratory tests. The study also showed a negative change in the average daily bowel movement frequency during the treatment period in both arms, which indicates improvement. Another result showed that 33.3% of the participants in each arm of the study recorded fecal incontinence during the treatment period.

In terms of AEs, no cases of mortality were recorded in both arms of the study. Among the participants who received eluxadoline 100 mg with BAM evidence, 91.67% of participants experienced AEs, the most prominent being hypothyroidism, abdominal pain, flatulence, nausea, constipation, diarrhea, dry mouth, vomiting, dizziness, and anxiety. Among the participants who received eluxadoline 100 mg without BAM evidence, 58.33% recorded symptoms, which included abdominal pain, flatulence, abdominal distention, nausea, constipation, renal hemorrhage, and hypotonia [12].

Efficacy, safety, and tolerability of JNJ-27018966 (eluxadoline) in the treatment of irritable bowel syndrome with diarrhea

This study is designed to determine the efficacy, safety, and tolerability of different doses of JNJ-27018966 (eluxadoline) compared with placebo in the treatment of patients with IBS-D. The study included 807 participants who were enrolled in a 12-week treatment period for the duration of one year. Of these patients, 18 were excluded from all datasets due to potential scientific misconduct, which left 789 patients to be included. The study was a randomized, double-blind, placebo-controlled, parallel-group, dose-ranging, and quadruple-masking study. The inclusion criteria included adult patients aged 18-65 years who had a diagnosis of IBS by the Rome III criteria with a subtype of diarrhea. Female patients must be postmenopausal, surgically sterile, abstinent, or if sexually active, must be on birth control. There were five arms included in this study. Patients were treated with eluxadoline 5 mg tablets in the first arm, eluxadoline 25 mg tablets in the second arm, eluxadoline 100 mg tablets in the third arm, and eluxadoline 200 mg tablets in the fourth arm. In the fifth arm, participants were given eluxadoline placebo-matching tablets. In each arm, participants were instructed to take the tablets orally BID for up to 12 weeks. The primary outcome measured the percentage of participants who were composite responders based on improvements from baseline in daily worst abdominal pain and daily stool consistency scores at week four.

The results showed that there is a difference when taking different doses of eluxadoline when measuring the percentage of participants who completed at least five out of seven days with diaries during the interval of interest and met both of the following criteria: (1) average daily pain response scores over the past week improved by at least ≥30% and at least two points as compared with the baseline average pain score (average of daily worst abdominal pain the week prior to randomization); (2) Bristol Stool Scale (BSS) score of 3 or 4 on 66% of reported days in the past week. At 5 mg, it had a p-value of 0.052, while at 25 mg, it had a p-value of 0.041, and at 100 mg, it had a p-value of 0.090. The study also measured the percentage of participants with a response based on participants achieving prespecified improvement in symptoms for at least 50% of the time over the treatment period. The results showed that participants who received 200 mg tablets BID in arm 4 had the highest percentage, with 30.6%, compared to 19.0% for participants who received placebo treatment.

In terms of AEs, no cases of mortality were recorded. Among the AEs, one out of 159 participants in the placebo arm reported anxiety and depression. One out of 105 participants who received eluxadoline 5 mg experienced non-cardiac chest pain. Three out of 170 participants who received eluxadoline 25 mg experienced AEs, which included pancreatitis, pneumonia, and major depression. One out of 165 participants who received eluxadoline 100 mg reported acute pancreatitis. Three out of 172 participants who received eluxadoline 200 mg reported events including alcoholic pancreatitis, acute pancreatitis, and perforated appendicitis. Every group reported gastrointestinal disorders, sinusitis, dizziness, and headaches [13].

Efficacy, safety, and tolerability of eluxadoline in the treatment of participants with diarrhea-predominant irritable bowel syndrome (IBS-D)

The purpose of this study is to determine the efficacy, safety, and tolerability of different doses of eluxadoline (JNJ-27018966) compared with placebo in the treatment of participants with IBS-D. The study enrolled 1146 participants after screening 3356 people, and out of all those participants, 250 completed arm 1, 264 completed arm 2, and 273 completed arm 3. The treatment period was 26 weeks over a duration of one year and six months. The study was randomized with a parallel assignment model and was triple masked. The inclusion criteria included participants aged 18-80 years old who had a diagnosis of IBS with a subtype of diarrhea defined by the Rome III criteria and had a colonoscopy performed. Female patients must be postmenopausal, surgically sterile, abstinent, or if sexually active, must be on birth control. The participants were divided into three arms. Patients were given eluxadoline 75 mg tablets in the first arm, eluxadoline 100 mg tablets in the second arm, and eluxadoline placebo-matching tablets in the third arm. In each arm, participants were instructed to take the tablets orally BID for up to 26 weeks of treatment, followed by placebo orally BID for the next four weeks of the blinded-placebo period. The primary outcome measured the percentage of participants who were composite responders based on improvements from baseline in daily worst abdominal pain and daily stool consistency scores.

The results showed that the percentage of participants who met the daily response criteria for at least 50% of the days during the interval of interest was 28.9% in the eluxadoline 75 mg arm, 29.6% in the eluxadoline 100 mg arm, and 16.2% in the placebo arm (p < 0.001). The study also measured the percentage of participants who were composite responders based on improvements from baseline in daily worst abdominal pain and daily stool consistency scores, which was 30.4% in the eluxadoline 75 mg arm, 32.7% in the eluxadoline 100 mg arm, and 20.2% in the placebo arm (p < 0.001).

In terms of AEs, no cases of mortality were recorded. Nine out of 379 participants in the eluxadoline 75 mg arm experienced AEs, the most prominent being angina pectoris, abdominal discomfort, gastric ulcer, acute pancreatitis, and chest pain. Among participants who received eluxadoline 100 mg, 14 out of 380 participants reported events including colitis ischemic, dyspepsia, hiatus hernia, acute pancreatitis, chest pain, hepatitis, and myasthenia gravis. Eight out of 381 participants who received the placebo reported events including acute coronary syndrome, coronary artery disease, and nephrolithiasis [14].

Efficacy, safety, and tolerability of eluxadoline in the treatment of participants with diarrhea-predominant irritable bowel syndrome (IBS-D)

This study determined the efficacy, safety, and tolerability of different doses of eluxadoline (JNJ-27018966) compared with placebo in the treatment of participants with IBS-D. The study included 1282 participants, and only 783 completed the study. Among them, 257 participants completed arm 1, 257 completed arm 2, and 269 completed arm 3. The duration of the study was two years, during which the treatment period was 52 weeks. It was a randomized, parallel assignment, triple-masked study. The inclusion criteria included participants with a diagnosis of IBS with a subtype of diarrhea defined by the Rome III criteria, and who had a colonoscopy performed. Female patients must be postmenopausal, surgically sterile, abstinent, or if sexually active, must be on birth control. Patients were given eluxadoline 75 mg tablets in the first arm, eluxadoline 100 mg tablets in the second arm, and eluxadoline placebo-matching tablets in the third arm. In each arm, participants were instructed to take the tablets orally BID for up to 52 weeks. The primary outcome measured the percentage of participants who were composite responders based on improvements from baseline in daily worst abdominal pain and daily stool consistency scores.

The results of the study showed that the percentage of participants who showed improvement was 23.0% in arm 1, 25.1% in arm 2, and 17.1% in arm 3. The eluxadoline 75 mg arm recorded a p-value of 0.014, while the eluxadoline 100 mg arm recorded a p-value of 0.004. The study also measured the percentage of participants who were composite responders based on improvements from baseline in daily worst abdominal pain and daily stool consistency scores, which was 23.4% in arm 1, 29.3% in arm 2, and 19.0% in arm 3. The eluxadoline 75 mg arm recorded a p-value of 0.112, and the eluxadoline 100 mg arm recorded a p-value < 0.001.

In terms of AEs, no cases of mortality were recorded. In the eluxadoline 75 mg arm, 5.84% of participants experienced AEs, the most prevalent being angina pectoris, myocardial infarction, abdominal pain, pancreatitis, chest pain, thyroid neoplasm, and abortion. Among the participants who received eluxadoline 100 mg, 5.64% of patients reported events including acute myocardial infarction, angina pectoris, stress cardiomyopathy, abdominal pain, colitis, acute pancreatitis, and asthma. Among the participants who received placebos, 3.75% of participants reported AEs, including papilledema, hemorrhoids, liposarcoma, and asthma. Every group reported gastrointestinal disorders as well as upper respiratory tract infections [15].

Effect of Lactobacillus plantarum 299v on symptoms of irritable bowel syndrome

This study examined whether supplementation with a probiotic, *Lactobacillus plantarum* 299v, demonstrates symptomatic efficacy in patients with IBS. The study included 81 participants, and only 65 of them completed the study. Forty participants completed arm 1 of the study and 25 participants completed arm 2. The duration of the study was 29 months, during which the treatment period was 12 weeks. The study design was randomized with parallel assignment and double masking. The inclusion criteria included participants who fulfilled the Rome II criteria for IBS, had at least one colonoscopy within the last three years, and were aged 18 years or older. The study divided participants into two arms. In the first arm, patients were treated with *Lactobacillus plantarum* 299v capsules, and in the second arm, they were treated with a placebo capsule filled with microcrystalline cellulose powder. The primary outcome measured the change in abdominal pain severity during the treatment period.

The results showed participants in the first arm scored 60.53 on a scale while participants in the second arm scored 54.06 on a scale (p < 0.05).

In terms of AEs, no cases of mortality were recorded. One out of 54 recipients of *Lactobacillus plantarum* 299v experienced a severe rash [16].

Study to evaluate the efficacy and safety of vibegron administered orally for 12 weeks to women with irritable bowel syndrome

This study evaluated the efficacy and safety of vibegron, a beta-3 adrenergic receptor (3-AR) agonist, in the treatment of pain associated with IBS due to IBS-D or mixed episodes of diarrhea and constipation (IBS-M). The study enrolled 222 women with IBS, and only 90 participants completed arm 1 of the study, and 99 participants completed arm 2. The duration of the study was 31 months, with a treatment period of 12 weeks. The study design was randomized with parallel assignment and triple masking. The inclusion criteria included female participants aged 18-70 years with a diagnosis of IBS with IBS-D or IBS with mixed episodes of diarrhea and constipation (IBS-M) according to the Rome IV criteria, who had undergone a colonoscopy and had no clinically significant findings on a physical examination or clinical laboratory tests that could interfere with study participation. In the first arm of the study, patients received a placebo, and in the second arm, they received vibegron 75 mg. In both arms, participants were instructed to take the tablet orally once daily for 12 weeks. Participants were divided into two groups based on their baseline abdominal pain intensity score (six versus six on a 0-to-10 numeric rating scale) and IBS subtype (IBS-D versus IBS-M). The primary outcome measured the number of participants with IBS-D who were abdominal pain intensity weekly responders.

The results showed that 27 out of 90 participants in arm 1 and 27 out of 99 participants in arm 2 recorded a decrease in the weekly average of "worst abdominal pain in the past 24 hours" scores of at least 30% compared with the baseline weekly average for at least 50% of the weeks assessed. The study also assessed the participants using the Global Improvement Scale. A total of 37 participants in arm 1 and 44 participants in arm 2 responded that their symptoms were either moderately relieved or significantly relieved.

In terms of AEs, no mortality cases were recorded. In the placebo arm, one out of 111 participants experienced hyperkalemia, and three experienced leukocyturia. In the vibegron 75 mg arm, two out of 111 participants experienced AEs, including COVID-19 and ectopic pregnancy. Both groups reported gastrointestinal disorders, infections, and headaches [17].

Does Welchol (colesevelam hydrochloride) improve colonic transit in diarrhea-predominant irritable bowel syndrome (D-IBS)? (Welchol)

This study set out to evaluate the effectiveness of Welchol (colesevelam hydrochloride) in improving colonic transit in patients who had IBS-D. The study enrolled 24 participants. The duration of the trial was four months. It was a randomized, parallel assignment, quadruple-masked study. The inclusion criteria included patients with IBS-D, aged 18-65 years, with no abdominal surgery (except appendectomy or cholecystectomy, as long as the patient's IBS-D symptoms preceded the cholecystectomy). The two arms of the study consisted of 12 participants who received colesevelam 1.875 g BID and 12 participants who received an inert capsule matching the study drug twice daily. The first primary outcome measured using the geometric center (GC) was colonial transit. The GC is the weighted average of counts in the different colonic regions. The scale ranges from one to five, where a high GC implies faster colonic transit. The second primary outcome measured was the half time for the ascending colon emptying.

The results showed that there was not any statistically significant difference between the two groups in terms of colonic transit time or in ascending colon emptying half time. In colon transit time, the first group was measured at 2.68 (0.32), while the second group was measured at 3.30 (0.33). For ascending colon emptying half time, the results for the first group were 18.85 hours, and the results for the second group were 14.9 hours.

In terms of AEs, no deaths were reported in either group. In addition, no serious AEs were reported in either group. However, all 12 participants in the first group did report AEs, including gastrointestinal disorders, headaches, and upper respiratory infections. Nine participants in the second group did report the same AEs [18].

Ability of Mayo Clinic high-performance liquid chromatography (HPLC) method to measure fecal bile acids

This study set out to evaluate the ability of colesevelam to reduce fecal BA and improve bowel function in patients with IBS-D, using Mayo's high-performance liquid chromatography (HPLC) method to measure fecal BA and demonstrate a response to colesevelam. The study enrolled 13 participants. The duration of the study was four months. The design of the study was a single group assignment with no masking. The inclusion criteria included patients with IBS-D. There were no restrictions on hospital anxiety and depression scores (HADS). They could have been male or female (after confirming that the female is not pregnant). The single-arm of the study included patients who received 1875 mg of colesevelam orally, twice daily, for 10 days. The primary outcome measured was the change in total 48-hour fecal BA from baseline in response to treatment with colesevelam. The stool 48-hour collections (for BAs) were collected during baseline before treatment, and then during days 9-10 of the 10 days of colesevelam dosing for fecal BAs. Total fecal BA was measured using HPLC/tandem mass spectrometry.

The result showed that after 10 days of treatment with colesevelam, there was a change in total 48-hour fecal BA from a baseline of 1662 uM to 3496 uM (p = 0.012).

In terms of AEs, no death was reported in either group. In addition, no serious AEs were reported. However, two participants did report constipation [19].

Study to evaluate the efficacy, safety, and tolerability of BOS-589 in the treatment of patients with diarrhea-predominant irritable bowel syndrome (IBS-D)

This trial set out to evaluate the efficacy, safety, and tolerability of BOS-589 in the treatment of patients with IBS-D. The trial enrolled 133 participants. The duration of the trial was 11 months. It was a randomized, parallel assignment, triple-masked study. The inclusion criteria included participants who had a diagnosis of IBS-D, recurrent abdominal pain occurring on average at least one day per week, and associated with changes related to defecation (frequency and consistency). In addition, the participants were negative for serum tissue transglutaminase immunoglobulin A (tTG-IgA) antibody. The trial had three arms. The first arm was an experimental group of 39 people, where participants received a high dose of BOS-589 orally BID. The second arm was an experimental group of 46 people, where participants received a low dose of BOS-589 orally BID. The third arm was a placebo comparator group of 40 people, where participants received a matching placebo orally BID. The primary outcome measured was the 24-hour worst abdominal pain on day 29 compared to baseline (averaged over the week prior to each respective time point).

The results showed that the first group that received a high dose of BOS-589 BID had a 24-hour worst abdominal pain score on day 29 of -1.64 (SD = 1.684) compared to the baseline score. The group that received a low dose of BOS-589 had a score of -2.30 (SD = 1.933) compared to the baseline score. The third group that received a placebo had a score of -1.69 (SD = 1.783) compared to the baseline score (p = 0.3776).

In terms of AEs, no deaths were reported in either group. In addition, no serious adverse effects were reported. However, the trial participants did have other adverse effects. The group that received a high dose of BOS-589 BID had 23 out of 39 participants that experienced AEs; the most prevalent were gastrointestinal disorders, infections and infestations, and dizziness. The group that received a low dose of BOS-589 BID had 23 out of 46 participants experience adverse effects, including gastrointestinal disorders, infections, and infestations. The third group that received a placebo had 15 out of 40 participants who experienced adverse effects, including gastrointestinal disorders and nervous system disorders [20].

Fecal microbiota transplantation for the treatment of diarrhea-predominant irritable bowel syndrome

This study set out to evaluate the effectiveness of fecal microbiota transplantation (FMT) for the treatment of IBS-D. The study enrolled 48 participants. The duration of the trial was 29 months. It was a randomized, crossover assignment, quadruple-masked study. The inclusion criteria included patients who were 19-65 years old, diagnosed with IBS-D, patients who had persistent symptoms despite conventional therapy, and patients who had a negative workup for celiac disease. The first arm of the study included 25 patients who took FMT capsules containing extensively screened donor stool. All of them took 25 FMT capsules on three consecutive days. The second arm of the study consisted of 23 patients who took placebo capsules that did not contain a donor’s stool or any active drug, and all of them took 25 FMT capsules on three consecutive days. At 12 weeks in the FMT arm, the patients crossed over to the other arm and vice versa, and that yielded the result of the 24 weeks. The primary outcome measured was "within and between-group comparisons of disease severity as determined by irritable bowel syndrome-symptom severity score (IBS-SSS)."

The results showed that the first group that received FMT capsules had a baseline score of 282 (SD = 65), by week 12, it was 221 (SD = 105), and by week 24, it was 208 (SD = 11). Meanwhile, the second group that received placebo capsules had a baseline score of 309 (SD = 64), by week 12, it was 236 (SD = 95), and by week 24, it was 157 (SD = 101).

In terms of AEs, no deaths were reported in either group. One serious AE was reported, i.e., a cholecystectomy in the placebo arm. However, it was noted that this was unrelated to the study, as this participant underwent cholecystectomy for acute cholecystitis two months after ingesting placebo capsules. Other AEs were also recorded. In the group that took FMT capsules, 23 participants reported gastrointestinal disorders, including abdominal pain, nausea, worsening diarrhea, and constipation. Of the 24 participants in the placebo group, eight also reported gastrointestinal disorders, including worsening diarrhea, bloating, abdominal pain, and gas [21].

Glutamine for the treatment of patients with irritable bowel syndrome (AT005291)

This study set out to evaluate the effectiveness of glutamine in the treatment of patients with IBS. The study enrolled 106 participants. The duration of the trial was 61 months. It was a randomized, parallel assignment, triple-masked study. The inclusion criteria were men and women aged 18-72 years old who developed post-infectious IBS-D, those who had increased intestinal permeability on the lactulose/mannitol permeability test, and the absence of alcohol or non-steroidal anti-inflammatory drugs ingestion for two weeks prior to inclusion in the study and throughout the study duration. The two arms of the study were 54 patients who took glutamine supplementation and 52 patients who took a placebo comparator in the form of whey protein powder. The primary outcome measure was the change in the Irritable Bowel Symptom Severity Scale (IBS-SS) from baseline to eight weeks after the conclusion of therapy. The IBS-SS scale ranges from 0 to 500 (worst), where a decrease of 50 or greater in the IBS-SS is considered a positive response.

The results showed that the group that received glutamine supplementation had a score of 181. The group that received a placebo in the form of a whey protein powder had a score of 301. Intestinal permeability was among the secondary outcomes measured. The glutamine group had a score of 0.05, while the whey protein group had a score of 0.10 (unit of measure: lactulose/mannitol). Another secondary outcome measured was stool frequency. Here, the glutamine group had 2.9 stools per day, compared to the whey protein group, which had 5.3 stools per day. In terms of AEs, none were reported in the study [22].

Mesalamine granules for irritable bowel syndrome (IBS) with diarrhea

The goal of this trial was to find the daily dose of mesalamine granules that would provide adequate relief from IBS symptoms with diarrhea. The study enrolled 148 participants. The duration of the study was 13 months. This was a phase two, randomized, placebo-controlled, double-blind, multicenter, 12-week study. The inclusion criteria included men and women diagnosed with IBS confirmed by the Rome III criteria and ≥3 average daily scores of bloating or abdominal pain with no relief in the past seven days on the first day of screening and on the day of randomization. The first arm had 41 patients on 1500 mg of mesalamine. The second arm had 47 patients on 750 mg of mesalamine and the third arm had 50 patients on the placebo capsule. It must be noted that only 138 out of the 148 people who completed the trial were on 1500 mg of mesalamine, 40 were on 750 mg, and 50 were on placebo. The primary outcome was the number of months that subjects were monthly responders to both IBS-related abdominal pain and stool consistency during the entire three-month time frame of this trial. "Monthly responders" are subjects who are weekly responders in both abdominal pain and stool consistency for at least two out of four weeks. A proportional odds model against the placebo was used to compare the number of months that the subjects were responders.

The results showed that for 1500 mg mesalamine users, an odds ratio of 2.232 was found (p = 0.0327), showing statistically significant results that 1500 mg of mesalamine improves abdominal pain and stool consistency compared to the placebo. Of the 51 patients, 18 patients responded in zero months, nine patients responded in one month, 13 patients responded in two months, and 11 patients responded in three months compared to the 50 placebos, in which 28 patients responded in zero months, eight patients responded in one month, six patients responded in two months, and eight patients responded in three months. For the 750 mg mesalamine users, an odds ratio of 1.148 (p = 0.7260) was found, showing no statistically significant difference between 750 mg mesalamine users and the placebo in monthly responders.

In terms of AEs, no deaths were reported. One notable AE was reported in the 750 mg mesalamine arm, an incision site abscess, and intervertebral disc degeneration in the same patient. A total of 29% of the 1500 mg mesalamine arm reported other AEs such as gastrointestinal disorders, infections, and headaches. A total of 19% of the 750 mg mesalamine arm, as well as 14% of the placebo arm, exhibited the same AEs [23].

Efficacy of mesalamine in diarrhea-predominant irritable bowel syndrome (dIBS)

The purpose of this study was to see if giving the anti-inflammatory drug mesalamine to patients with IBS-D would help improve their symptoms of diarrhea, bloating, and abdominal pain. The study enrolled seven participants and had a duration of two years. It was a randomized, double-blind, cross-over study with a 12-week time period from baseline. Inclusion criteria included patients aged 18-65 years with a Functional Bowel Disorder Severity Index (FBDSI) score of 37 or higher, a normal complete blood count, normal liver enzyme level, no stool infectious diarrhea, and no history of chronic liver disease, heart disease, pulmonary disease, or renal disease. Patients with a history of chronic liver disease, heart disease, pulmonary disease, or renal disease were excluded. It also excluded patients on steroids, antacids, warfarin, or chronic pain conditions other than fibromyalgia. Five people each received the drug mesalamine (1500 mg) for 12 weeks, then a washout time of three weeks prior to crossing over to the placebo arm (1500 mg of sugar pills) for another 12 weeks. Alternatively, two people received the placebo for 12 weeks, then a washout for three weeks prior to crossing over to the drug arm for another 12 weeks. One patient from the first group did not complete the study before having his or her placebo measurement. The primary outcome measure was changed in gastrointestinal scores (GIS) between baseline time and after a 12-week intervention with mesalamine or placebo. To make the GIS, patients rated the severity of their GI symptoms. The GIS scale goes from 1 to 7, with 1 being the worst and 7 being the best score, showing improvement in symptoms.

In this study, seven (the total mesalamine patients independent of the order) and six (the total placebo patients independent of the order) patients were analyzed before the washout. For the mesalamine users, a +2.72 improvement (p = 0.001) on the seven-point GIS scale was recorded, while a +2.22 improvement (p = 0.08) was recorded for the placebo users with the seven-point GIS scale. In terms of AEs, neither deaths nor any major or minor AEs were recorded in this study [24].

The effect of long-acting mesalamine on post-infective irritable bowel syndrome - a pilot study (mesalamine)

The purpose of this study was to evaluate the effects of long-acting mesalamine (Lialda) in patients with post-infective IBS (PI-IBS). It enrolled 61 participants over a duration of nine years. It was a triple-blind, randomized, parallel assignment study. Inclusion criteria were men and women aged 18-75 years with the Rome III criteria for IBS and negative for celiac disease and inflammatory bowel disease. Arm 1 consisted of 30 patients who started on an inactive placebo, but four did not complete the study. Arm 2 consisted of 31 patients who started on 1200 mg of mesalamine, but three did not complete the study. The primary outcome measured was the change in average overall bowel symptom score (BSS) after an eight-week treatment period. The BSS score is calculated from the sum of BSS items of abdominal pain severity, bloating severity, diarrhea severity, constipation severity, and how satisfied you are with your bowel habits and is measured on a scale of 0% to 100%, with 0 being not severe and 100 being very severe.

A mean BSS improvement of -4 (standard error of 3) was measured in the placebo group. A mean BSS improvement of -13 (standard error of 3) was measured in mesalamine patients.

In terms of AEs, no mortalities or serious AEs were reported. Ten out of 33 placebo patients had complications, most frequently GI disorders and chest pain. Of the mesalamine patients, 16/31 patients had complications, mainly GI disorders, infections, and headaches [25].

Randomized, double-blind, placebo-controlled phase 2 trial of Bekinda (ondansetron 12 mg bimodal release tablets) for diarrhea predominant irritable bowel syndrome (IBS-D)

This study set out to analyze the stool consistency response rate (SCRR) in patients with Bekinda. It enrolled 127 participants and had a duration of a year. It was a randomized, double-blind, placebo-controlled, two-arm, parallel-group study. Patients underwent a two-week observation period during which stool consistency and frequency data and symptom data were collected. Patients were then randomized to 75 participants on RHB-102 (12 mg) as well as 42 participants on placebo. The inclusion criteria consisted of male and female patients aged 18 years and above who met FDA guidance and the Rome III criteria for IBS-D, with an average worst daily pain intensity of 3.0 for each of the two baseline weeks, adequate hematologic function, and adequate liver and renal function. The primary outcome was the overall SCRR.

Results showed that for the 75 patients on Bekinda, 42 patients were weekly stool consistency respondents (56% of the 75 patients) and 33 patients were not (44%). Of the 52 patients on placebo, 18 patients were weekly stool consistency respondents (35.3% of the 52 patients) and 33 were not (64.7%). A comparison of the two arms (p = 0.036) was done, showing a statistically significant improvement in SCRR when taking Bekinda.

In terms of AEs, no deaths were reported in the study. However, 21.33% of the Bekinda patients reported GI disorders such as flatulence and constipation, and a further 5.33% reported UTIs. The placebo group showed similar events, but at a different prevalence (3.92% showed GI disorders and 1.96% showed UTIs) [26].

The efficacy of PX0612 in the treatment of irritable bowel syndrome

This study investigated the efficacy of PX0612 in the treatment of IBS. The study enrolled 50 participants. They were divided into 25 patients (23 of whom completed the study) on a veggie capsule that contains PX0612 and 25 patients (21 of whom completed the study) on dicalcium phosphate placebos. The duration of the study was 31 months. It was a randomized, double-blind, placebo-controlled clinical trial. The inclusion criteria consisted of 18-65-year-old men and women with mild to moderate IBS-D on the FBDSI index. The primary outcome measure was the mean change in the frequency of bowel movements (stool frequency - bowel movements/day) between the "intervention" group and the "placebo" group over the study period. A higher mean score indicates a better outcome and a greater reduction in bowel movements per day, which is a positive change.

Results found a 0.46 BM/day improvement was seen in the PX0612 patients compared to the 0.21 BM/day improvement in the placebo patients.

In terms of AEs, no serious events or deaths were recorded, but 28% of the PX0612 patients had events, all of which were GI disorders such as heartburn. Meanwhile, 60% of the placebo group had events including GI disorders but also thyroiditis, back pain, and headaches [27].

A study of MD-7246 to treat abdominal pain in patients with diarrhea-predominant irritable bowel syndrome

This study set out to evaluate the safety and tolerability, the treatment effect on abdominal pain, and dose-response of MD-7246 administered orally to patients with IBS-D. The study enrolled 515 participants, and the duration of the study was 10 months. This was a phase two, randomized, double-blind, placebo-controlled, parallel-group, dose-range-finding study. The inclusion criteria included patients meeting the Rome IV criteria for diagnosis of IBS-D, aged 18 years or older. Patients also had to maintain a minimum level of compliance with their daily diary and use contraception for female patients of childbearing potential. This study included a pretreatment period of 14-21 days immediately before randomization. During this period, participants underwent symptomatic assessments in an electronic diary (eDiary); those who satisfied all entry criteria based on these assessments then entered the treatment period and were randomized to one of four treatments: MD-7246 300, 600, or 1200 μg or placebo in a 1:1:1:1 ratio. Of the 515 participants in the pretreatment period, 127 were not randomized. There were four arms with 97 patients each. Arm 1 had participants on four placebo oral tablets daily, while arm 2 had participants on one MD-7246 300 μg tablet and three placebo tablets. Arm 3 had participants on two MD-7246 300 μg tablets and two placebo tablets. Arm 4 participants were on four MD-7246 300 μg tablets and no placebo tablets. The primary outcome measure was the change from baseline in abdominal pain at its worst on a Numeric Rating Scale (NRS) through the treatment period (eDiary logs, using an 11-point NRS, where 0 is anchored with "no abdominal pain" and 10 is anchored with "worst possible abdominal pain").

Results showed that the change in NRS for the placebo group was -2.01, -1.93 for the 300-μg group, -1.58 for the 600-μg group, and -1.95 for the 1200-μg group. The p-values compared to placebo for the respective treatment groups were 0.7467, 0.0941, and 0.8098.

In terms of AEs, no deaths were reported in the study. However, ectopic pregnancy was recorded in the 1200 μg arm. However, other adverse effects were reported in all the arms. Those most prevalent included GI disorders, infections, and headaches. The prevalence of AEs rose with the increasing concentration of the drug. It rises from 28.87% in the 300-μg arm to 43.30% in the 600-μg arm, and finally 53.61% in the 1200-μg arm [28].

Table 1 summarizes the results of all the mentioned clinical trials.

**Table 1:** Summary of the clinical trials IBS-D: irritable bowel syndrome with diarrhea; BSFS: Bristol Stool Form Scale; BSS: Bristol Stool Scale; IBS-SSS: IBS symptom severity scale; GIS: gastrointestinal symptom; BID: twice per day; FMT: fecal microbiota transplantation; SCRR = stool consistency response rate; NRS: Numeric Rating Scale.

Title	Agent used	Primary outcome	Reference
Irritable bowel syndrome with diarrhea (IBS-D) rifaximin re-treatment study (TARGET3)	Rifaximin	The proportion of patients who responded to treatment was 32.6% for the rifaximin group (p = 0.0232)	NCT01543178 [6]
Rifaximin 3 times/day (TID) for non-constipation irritable bowel syndrome (IBS) (TARGET 1)	Rifaximin	Subjects who had relief of IBS symptoms was 40.8% for the rifaximin group (p = 0.01)	NCT00731679 [7]
Rifaximin 3 times/day (TID) for non-constipation irritable bowel syndrome (IBS) (TARGET 2)	Rifaximin	Subjects who had relief of IBS symptoms was 40.6% for the rifaximin group (p = 0.03)	NCT00724126 [8]
12-week efficacy and safety study of ibodutant in women with irritable bowel syndrome with diarrhea (IBS-D) (IRIS-3)	Ibodutant	Response for abdominal pain intensity and stool consistency was 35.7%	NCT02107196 [9]
Ibodutant for relief of irritable bowel syndrome with diarrhea (IBS-D) (IRIS-2)	Ibodutant	Subjects who had relief according to the 75% rule were 45 participants in the ibodutant 1 mg arm, 46 participants in the ibodutant 3 mg arm, and 55 participants in the ibodutant 10 mg arm (p < 0.05)	NCT01303224 [10]
Efficacy of eluxadoline in the treatment of irritable bowel syndrome with diarrhea in patients with inadequate control of symptoms with prior loperamide use	Eluxadoline	The percentage of participants who were composite responders from baseline to 12 weeks was 22.7% for the eluxadoline 100 mg group (p = 0.0022)	NCT02959983 [11]
Eluxadoline bile acid malabsorption (BAM) study	Eluxadoline	The results in both arms of the study showed a negative change from baseline in average (BSFS), which indicates improvement as a result of treatment. When assessing the number of participants who experienced potentially clinically significant changes in laboratory tests, the first arm of the study in which participants had evidence of bile acid malabsorption showed no change in laboratory tests. In the second arm of the study, the participants had no evidence of bile acid malabsorption	NCT03441581 [12]
Efficacy, safety, and tolerability of JNJ-27018966 (eluxadoline) in the treatment of irritable bowel syndrome with diarrhea	Eluxadoline	Completed at least 5 out of 7 days with diaries during the interval of interest and met both of the following criteria: (1) average daily pain response scores over the past week improved by at least ≥30%; (2) BSS score of 3 or 4 on 66% of reported days in the past week, that there is a difference when taking different doses of eluxadoline. At 5 mg, it had a p-value of 0.052, while at 25 mg, it had a p-value of 0.041, and at 100 mg, it had a p-value of 0.090	NCT01130272 [13]
Efficacy, safety, and tolerability of eluxadoline in the treatment of participants with diarrhea-predominant irritable bowel syndrome (IBS-d)	Eluxadoline	Participants who met the daily response criteria for at least 50% of the days during the interval of interest were 28.9% in the eluxadoline 75 mg arm, 29.6% in the eluxadoline 100 mg arm, and 16.2% in the placebo arm (p < 0.001)	NCT01553747 [14]
Efficacy, safety, and tolerability of eluxadoline in the treatment of participants with diarrhea-predominant irritable bowel syndrome (IBS-d)	Eluxadoline	Participants who showed improvement was 23.0% in arm 1, 25.1% in arm 2, and 17.1% in arm 3. The eluxadoline 75 mg arm had p = 0.014, while the eluxadoline 100 mg arm had p = 0.004	NCT01553591 [15]
Effect of *Lactobacillus plantarum* 299v on symptoms of irritable bowel syndrome	*Lactobacillus plantarum* 299v	Participants in the first arm scored 60.53 on a scale while participants in the second arm scored 54.06 on a scale (p < 0.05)	NCT01886781 [16]
Study to evaluate the efficacy and safety of vibegron administered orally for 12 weeks to women with irritable bowel syndrome	Vibegron	Twenty-seven out of 90 participants in arm 1 and 27 out of 99 participants in arm 2 recorded a decrease in the weekly average of "worst abdominal pain in the past 24 hours" scores of at least 30% compared with the baseline weekly average for at least 50% of the weeks assessed	NCT03806127 [17]
Does Welchol (colesevelam hydrochloride) improve colonic transit in diarrhea-predominant irritable bowel syndrome (D-IBS)? (Welchol)	Welchol	No statistically significant difference between the two groups in terms of colonic transit time or in ascending colon emptying half time	NCT00911612 [18]
Ability of Mayo Clinic high-performance liquid chromatography (HPLC) method to measure fecal bile acids	Colesevelam	The participants showed a change in total 48-hour fecal bile acids from a baseline of 1662 uM to 3496 uM (p = 0.012)	NCT02111603 [19]
Study to evaluate the efficacy, safety, and tolerability of BOS-589 in the treatment of patients with diarrhea-predominant irritable bowel syndrome (IBS-D)	BOS-589	The participants who received a high dose of BOS-589 BID had a 24-hour worst abdominal pain score on day 29 of -1.64 (SD = 1.684) compared to the baseline score. The group that received a low dose of BOS-589 had a score of -2.30 (SD = 1.933) compared to the baseline score	NCT03977155 [20]
Fecal microbiota transplantation for the treatment of diarrhea-predominant irritable bowel syndrome	Fecal microbiota transplantation	The participants who received FMT capsules had a baseline score of 282 (SD = 65), and by week 12 it was 221 (SD = 105) and by week 24 it was 208 (SD = 11)	NCT02328547 [21]
Glutamine for the treatment of patients with irritable bowel syndrome (AT005291)	Glutamine	The group that received glutamine supplementation had a score of IBS-SSS 181	NCT01414244 [22]
Mesalamine granules for irritable bowel syndrome (IBS) with diarrhea	Mesalamine granules	The participants showed statistically significant results that 1500 mg of mesalamine improves abdominal pain and stool consistency (p = 0.0327)	NCT01177410 [23]
Efficacy of mesalamine in diarrhea-predominant irritable bowel syndrome (dIBS)	Mesalamine	For the mesalamine users, a +2.72 improvement (p = 0.001) on the 7-scale GIS was recorded	NCT01327300 [24]
The effect of long-acting mesalamine on post-infective irritable bowel syndrome- a pilot study (mesalamine)	Long-acting mesalamine	A mean BSS improvement of -4 (standard error of 3) was measured in the placebo group. A mean BSS improvement of -13 (standard error of 3) was measured in mesalamine patients	NCT01412372 [25]
Randomized, double-blind, placebo-controlled phase 2 trial of BEKINDA (ondansetron 12 mg bimodal release tablets) for diarrhea predominant irritable bowel syndrome (IBS-D)	Bekinda	Forty-two patients were weekly stool consistency respondents (56% of the 75 patients). A comparison of the two arms (p = 0.036) was found, showing a statistically significant improvement in SCRR when taking Bekinda	NCT02757105 [26]
The efficacy of PX0612 in the treatment of irritable bowel syndrome	PX0612	Results found a 0.46 BM/day improvement was seen in the PX0612 patients	NCT02431533 [27]
A study of MD-7246 to treat abdominal pain in patients with diarrhea-predominant irritable bowel syndrome	MD-7246	Results showed that the change in NRS for 300 μg group was -1.93 (p = 0.7467), -1.58 for the 600 μg group (p = 0.0941), and -1.95 for the 1200 μg group (p = 0.8098)	NCT03931785 [28]

## Conclusions

Research on IBS therapy has so far had some success in terms of symptomatic treatments. This is due to the fact that IBS has many alternative pathophysiologic pathways that can explain the symptoms accompanying the disease. Prior to the development of therapies, research focused on the pathophysiologic mechanisms underlying disease development, which aided in understanding how symptoms arise. Today, most of the therapeutic approaches for IBS-D type are targeted at the treatment of one symptom amongst many. This study set out to examine various new medicines in the treatment of IBS-D and summarize the findings. All the drugs were in a clinical trial stage to determine their effectiveness, safety, and duration of therapy and to determine the safe dose. The clinical trials for these drugs used different models to obtain the results, as well as different target groups. This research has summarized the findings of these major clinical trials to help aid readers in understanding the important results and identify new areas of improvement. Although some of these drugs have shown significant improvement in symptoms, we still have a long way to further understand and treat this illness that affects so many patients worldwide.
